# PD224 Evaluating Participant Satisfaction In Health Technology Assessment Reports: A Survey Study In Catalonia, Spain

**DOI:** 10.1017/S0266462324004434

**Published:** 2025-01-07

**Authors:** Laura Llinàs-Mallol, Carolina Moltó-Puigmartí, oan Segur-Ferrer, Jessica Ruiz-Baena, Edurne Gallastegui, Anna Godo Pla, Maria Dolors Estrada Sabadell, Rosa Maria Vivanco-Hidalgo

## Abstract

**Introduction:**

Developing a health technology assessment (HTA) report requires the involvement of relevant stakeholders, specifically healthcare professionals, patients, and industry representatives. Evaluating the satisfaction of participants in HTA reports is crucial to improving internal procedures and increasing the participation of stakeholders, who play a crucial role in adding knowledge and experience to the reports.

**Methods:**

We developed a nine-question survey to assess the satisfaction of healthcare professionals and patients involved in HTA reports. The questions evaluated global satisfaction with participating in the HTA report (scored 0 to 10), as well as with the information received to develop tasks within the report and the time given to perform them. Healthcare professionals (n=86), patients (n=3), and contributors to outcome prioritization (n=4) involved in 12 HTA reports developed in Catalonia, Spain were invited to participate.

**Results:**

Thirty-two contributors (34.4%) responded. Twenty-nine were healthcare professionals (33.7% response rate) and three were patients (100% response rate); 59.4 percent of respondents had only participated in one HTA report. Contributors rated their participation with 8.53 points, with external reviewers and patients being the most satisfied collaborators (9.33 and 9.00 points, respectively). Twenty-eight participants (87.5%) felt they had received enough information to develop their tasks in the report, while the other four (12.5%) would have preferred more specific information about the aims of the evaluation. All but one contributor was satisfied with the amount of time given to perform the required tasks.

**Conclusions:**

Collaborators in HTA reports were satisfied with their participation. Both the information provided about the tasks and the time provided to perform them were adequate. Nevertheless, the assessment team must always ensure that the aims of the evaluation are clear to everyone. We plan to evaluate the satisfaction of industry collaborators in the near future.

